# T cell receptor diversity evaluation to predict patient response to Ipilimumab in metastatic melanoma

**DOI:** 10.1186/2051-1426-2-S3-O8

**Published:** 2014-11-06

**Authors:** Michael Postow, Manuarii Manuel, Phillip Wong, Jianda Yuan, Marlene Noel, Anais Courtier, Nicolas Pasqual, Jedd D Wolchok

**Affiliations:** 1 Memorial Sloan Kettering Cancer Center, New York, NY, USA; 2 ImmunID, Grenoble, France; 3 Immune Monitoring Facility, Sloan Kettering Institute, New York, NY, USA

## Background

Ipilimumab blocks the immunologic checkpoint cytotoxic T lymphocyte antigen-4 (CTLA-4) and improves overall survival in patients with metastatic melanoma. Since only a subset of patients achieves benefit from ipilimumab, biomarkers that can predict patient outcome are needed. CTLA-4 blockade has been shown to affect the peripheral T cell receptor (TCR) pool, but whether diversity of the peripheral TCR repertoire is relevant as a biomarker for ipilimumab remains unknown.

## Methods

In this pilot study, we analyzed the baseline (pretreatment) peripheral blood TCR repertoire in 12 patients with metastatic melanoma treated with ipilimumab at 3mg/kg (investigator assessed clinical benefit, n = 4; non-clinical benefit, n = 8). TCR diversity was evaluated using a multi-N-plex polymerase chain reaction (PCR) assay which measures TCR combinatorial diversity between V and J genes from genomic DNA. The diversity of the TCR pool was studied through richness (observed V-J rearrangements) and clonality (evenness of the repertoire based on frequencies of specific V-J rearrangements). The Wilcoxon rank sum test was used to compare results between patients with clinical benefit and those without. A receiver operating characteristic (ROC) curve was used to determine an appropriate threshold for dichotomized analysis (i.e. low vs. high diversity richness/clonality). Association with benefit in the low vs. high groups was assessed through a Fisher's exact test. Overall survival was studied through log-rank analysis.

## Results

There was a significant difference in diversity richness (p = 0.033) and in clonality (p = 0.028) between patients with and without clinical benefit. Dichotomized analysis showed that none of the patients with low diversity richness (n = 0/5, p = 0.081) nor low clonality (n = 0/7, p = 0.01) achieved clinical benefit. 4 out of the 5 patients with a high clonality had clinical benefit. There was no significant difference in overall survival between patients with low vs. high diversity richness (p = 0.218) and clonality (p = 0.26).

## Conclusions

In a small group of melanoma patients treated with ipilimumab, TCR diversity in the peripheral blood at baseline was associated with patient outcomes. Further investigation is ongoing in larger cohorts of patients treated with novel immunologic checkpoint antibodies to explore these preliminary findings and determine whether TCR diversity can be used as a predictive biomarker in cancer immunotherapy.

## Consent

Written informed consent was obtained from the patient for publication of this abstract and any accompanying images. A copy of the written consent is available for review by the Editor of this journal.

